# Three-dimensional ultrasonography: Advancing the clinical evaluation of adolescent idiopathic scoliosis^☆^

**DOI:** 10.1016/j.ultsonch.2025.107391

**Published:** 2025-05-17

**Authors:** Xinyu Yang, Derun Di, Yanhong Lv, Zhe Liu, Xiaoxiong Wang, Xinzhi Zhang, Rongkun Xu, Luoran Wang, Yuze Wang, Suomao Yuan, Yonghao Tian, Qiang Yang, Xinyu Liu, Lianlei Wang

**Affiliations:** a Department of Orthopedics, Qilu Hospital of Shandong University, Jinan, Shandong, China; b Qilu Hospital, Cheeloo College of Medicine, Shandong University, Jinan, Shandong, China; c Department of Spine Surgery, Tianjin Hospital, Tianjin University, Tianjin, China; d School of Nursing and Rehabilitation, Cheeloo College of Medicine, Shandong University, Jinan, Shandong 250012, China

**Keywords:** Adolescent idiopathic scoliosis, Three-dimensional ultrasound, Spinal curvature, Cobb angle, Reliability, Validity

## Abstract

The clinical efficacy of 3D ultrasound for spinal curvature assessment was evaluated in adolescent idiopathic scoliosis (AIS) patients through comparative analysis with conventional radiography. A prospective cohort of 230 preoperative and 22 postoperative AIS patients was evaluated over a 24-month period. Spinal curvature assessments were performed using a standardized 3D ultrasound system (SCN801), with reliability quantified via intraclass correlation coefficients (ICC) and validity assessed through Spearman correlation analysis. Ultrasound measurements demonstrated good reliability (ICC > 0.70), particularly for transverse process (TP)-based angle measurements, which showed superior consistency (ICC > 0.80). Strong correlations were observed between radiographic Cobb angles and TP-derived ultrasound curve angles (UCA) in mild-to-moderate curves (R2 > 0.70), though correlations diminished in severe deformities (R2 > 0.6). Mean scanning time measured 37.3 ± 6.8 s, with diagnostic-quality images obtained in 81 % of initial acquisitions. Multivariate regression analysis identified thoracic kyphosis magnitude (B = 3.334, p = 0.012) and vertebral rotation severity (B = 1.862–2.209, p < 0.05) as significant predictors of measurement variability in thoracic and (thoraco) lumbar curves. The findings demonstrate that TP-based 3D ultrasound enables accurate radiation-free spinal assessments in mild-to-moderate AIS, with measurement accuracy significantly influenced by vertebral rotation and thoracic kyphosis severity. Despite reduced performance in complex deformities, the method demonstrates operational efficiency, diagnostic precision, and an enhanced safety profile, supporting its clinical utility as a screening alternative to conventional radiography.

## 1 Introduction

Adolescent idiopathic scoliosis (AIS) is a complex three-dimensional spinal deformity characterized by structural lateral curvature of the spine in the coronal plane with rotational abnormalities in the axial plane [[1], [2], [3]]. This condition affects approximately 1 % to 4 % of adolescents during pubertal growth spurts, potentially leading to severe clinical consequences including significant trunk deformities, impair cardiopulmonary function, and reduced life expectancy in severe cases [4,5]. The insidious nature of early-stage AIS presentation underscores the critical need for developing reliable screening modalities to facilitate timely diagnosis and intervention [6,7]. Furthermore, as the scoliosis rapidly progresses during adolescence, regular monitoring of curve progression in AIS patients is essential [8].

While posteroanterior radiographs remain the gold standard for quantifying Cobb angles in AIS diagnosis and progression monitoring [9]. The cumulative radiation doses from repeated examinations paradoxically elevate lifetime attributable cancer risk, particularly concerning for adolescent females requiring longitudinal follow-up [10]. Although low-dose biplanar radiography (EOS imaging) reduces radiation exposure while maintaining image quality, significant barriers including prohibitive equipment costs, specialized facility requirements and limited accessibility in developing regions continue to restrict its clinical penetration [11]. Furthermore, conventional screening modalities like the Adams forward bend test and scoliometer measurements exhibit persistent limitations in diagnostic accuracy, limiting their diagnostic reliability as primary evaluation tools [[12], [13], [14]].

Ultrasound imaging has established itself as an indispensable diagnostic modality in modern medicine, leveraging its non-invasive, real-time visualization, and cost-efficiency to enable widespread clinical applications ranging from obstetric monitoring to cardiovascular and abdominal assessments [15,16]. Recent advancements in 3D ultrasound have positioned this non-ionizing diagnostic modality as a promising alternative for AIS evaluation. Mounting studies have demonstrated its measurement reliability for Cobb angle quantification in standard AIS populations, while achieving comparable reliability to conventional radiography [[17], [18], [19], [20], [21], [22]]. Nevertheless, broader implementation of this methodology remains constrained by insufficient sample diversity and undetermined generalizability across heterogeneous clinical populations. Additionally, there is a lack of analysis on imaging-related factors that influence measurement accuracy, a critical knowledge gap that hinders evidence-based optimization of medical device configurations and iterative development cycles.

The identification of a portable, cost-effective, and radiation-free diagnostic modality holds critical significance for the diagnosis and monitoring of AIS. Therefore, the aim of this study is to systematically evaluate method diagnostic reproducibility and clinical validity across a heterogeneous AIS cohort stratified by different measurement methods, different severity, different age, and postoperative AIS, and investigate imaging-related factors influencing measurement accuracy.

## 2 Materials and methods

### 2.1 Study population

**Fig. 1:**
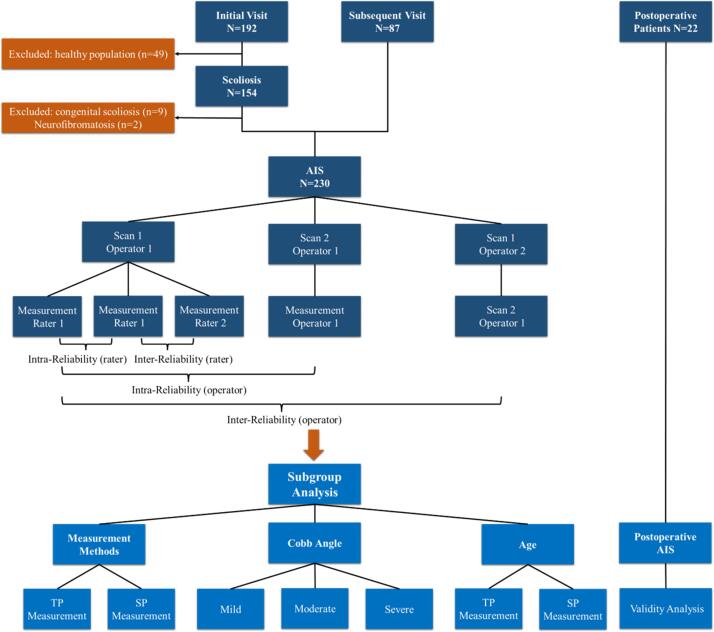
Schematic of the study design.

This prospective cohort study received ethical approval from the Institutional Review Board of Qilu Hospital, Shandong University. A total of 252 AIS patients (230 preoperative cases; 22 postoperative cases) were consecutively enrolled between January 2022 and February 2024 (Fig. 1).

Inclusion criteria: (1) Patients diagnosed with AIS; (2) Informed consent was obtained from all patients and their parents; (3) Patients were required to undergo both ultrasound scan and X-ray imaging on the same day. Exclusion criteria were: (1) Patients younger than 10 years or older than 18 years; (2) Presence of other spinal deformities, such as congenital scoliosis, neuromuscular scoliosis, or spinal tumors; (3) The presence of winged scapula in patients affected the smooth execution of the scanning process; (4) Allergy to ultrasound gel; (5) Significant comorbidities that may affect participation in the study or outcomes.

### 2.2 Three-dimensional ultrasound imaging

Ultrasound images were acquired using a three-dimensional ultrasound imaging system (Model SCN801, Telefield Medical Imaging Ltd., Hong Kong) [23]. The trained clinicians operated the ultrasound probe and stably scanned the spinal images from S1 to T1 at a speed of 1 cm/s, while participants maintained a neutral standing posture with forward gaze to ensure optimal cervicothoracic alignment and minimize motion artifacts. Post-acquisition, the system proprietary software automatically reconstructed volumetric datasets into two-dimensional coronal plane projections, with the ultrasound curvature angle (UCA) quantified through automated detection of all identifiable transverse process (TP) and spinous process (SP) anatomical landmarks.

### 2.3 Reliability and validity

The TP and SP measurement protocols were validated for reliability and validity using Brink et al. methodology [18]. Reliability assessment comprised intra-observer (Operator 1 repeated scans with standardized repositioning) and inter-observer (Operator 1 versus 2 comparisons) analyses for scan acquisition consistency, complemented by intra-observer (Rater 1 blinded 7-day interval remeasurements) and inter-observer (Rater 1 versus 2 evaluations) reproducibility testing. Concurrent validity was established through Pearson correlation comparing UCA with radiographic Cobb angle measurements across the cohort.

Patients were stratified into clinically stratified subgroups: (1) Scoliosis severity − mild (Cobb ≤ 20°), moderate (20° < Cobb < 35°), and severe (Cobb ≥ 35°); (2) Age-specific cohorts: screening (10–12 years) versus progression (≥ 13 years) groups. Postoperative methodological validation incorporated longitudinal ultrasound-radiograph correlation analysis. Multivariable linear regression identified covariates influencing measurement reliability, including body mass index, axial vertebral rotation, and thoracic hypokyphosis.

### 2.4 Statistical analysis

All statistical analyses were performed using SPSS software (Version 26.0, Chicago, IL, USA). Descriptive statistics included mean ± SD and ranges. Reliability assessment employed intraclass correlation coefficients (ICC) with two-way random absolute agreement, interpreted per established criteria: 0.80–1.00 (excellent), 0.60–0.79 (moderate), and ≤ 0.60 (poor). Consistency between measurement modalities was quantified using Pearson correlation coefficients. Multivariate linear regression modeling (adjusted for age and gender) evaluated the independent effects of obesity (BMI ≥ 30 kg/m^2^), vertebral rotation (Nash-Moe grade I + ), and thoracic kyphosis (> 40°) on ultrasound measurement accuracy, with standardized β coefficients and 95 % confidence intervals calculated to assess variable contributions.

## 3 Results

### 3.1 Assessing reliability

Baseline parameters for demographic characteristics are shown in Table 1. All the reliability of UCA is summarized in Table 2 and Table 3. Both TP and SP measurement protocols demonstrated clinically acceptable reliability (ICC > 0.70) across AIS severity strata, though revealed a statistically significant progressive reduction in ICC values with increasing curvature magnitude. For UCA based on the SP angle, the intra- and inter-rater/operator ICC also showed good reliability (ICC > 0.70), lower than those of the TP angle (ICC > 0.80).

**Table 1:** Demographic and clinical information of preoperative patients.

**Items**	**Values (mean** ± **SD)**
**Number of patients**	
Total	230
With one curve	83
With two curves	147
**Age (years)**	13.85 ± 3.48
**Gender (female: male）**	157: 73
**Body mass index (kg/m2)**	18.15 ± 3.37
**Scan time (seconds)**	37.30 ± 3.54
**Location of scoliosis**	
Thoracic curve	
Average Cobb angle (degrees)	25.90 ± 13.20°
Mild	74 (42.04 %)
Moderate	61 (34.66 %)
Severe	41 (23.30 %)
(Thoraco) Lumbar curve	
Average Cobb angle (degrees)	25.66 ± 11.81°
Mild	80 (39.80 %)
Moderate	81 (40.30 %)
Severe	40 (19.90 %)

**Table 2:** Three-dimensional ultrasound assessment of thoracic curvature angle reliability.

**Ultrasound Imaging**	**Thoracic curve**			
	**Total**	**Mild**	**Moderate**	**Severe**
**TP Intra-rater**	0.96 (0.92–0.97)	0.96 (0.94–0.98)	0.95 (0.91–0.98)	0.92 (0.86–0.98)
**TP Inter-rater**	0.93 (0.89–0.96)	0.93 (0.83–0.92)	0.91 (0.84–0.91)	0.90 (0.84–0.98)
**TP Intra-operator**	0.90 (0.86–0.92)	0.90 (0.88–0.94)	0.88 (0.84–0.92)	0.85 (0.80–0.91)
**TP Inter-operator**	0.87 (0.83–0.90)	0.87 (0.85–0.91)	0.85 (0.80–0.90)	0.82 (0.78–0.88)
**SP Intra-rater**	0.94 (0.92–0.97)	0.94 (0.93–0.98)	0.92 (0.90–0.97)	0.90 (0.87–0.96)
**SP Inter-rater**	0.92 (0.88–0.96)	0.92 (0.90–0.97)	0.89 (0.86–0.95)	0.86 (0.83–0.92)
**SP Intra-operator**	0.88 (0.84–0.91)	0.85 (0.81–0.89)	0.82 (0.77–0.87)	0.80 (0.74–0.84)
**SP Inter-operator**	0.86 (0.82–0.89)	0.80 (0.75–0.86)	0.75 (0.70–0.82)	0.70 (0.65–0.78)

**Table 3:** Three-dimensional ultrasound assessment of (thoraco) lumbar curvature angle reliability.

**Ultrasound Imaging**	**(Thoraco) Lumbar curve**			
	**Total**	**Mild**	**Moderate**	**Severe**
**TP Intra-rater**	0.94 (0.86–0.95)	0.92 (0.86–0.95)	0.91 (0.83–0.95)	0.90 (0.84–0.99)
**TP Inter-rater**	0.91 (0.85–0.93)	0.90 (0.83–0.93)	0.89 (0.81–0.93)	0.88 (0.80–0.93)
**TP Intra-operator**	0.88 (0.81–0.90)	0.86 (0.78–0.88)	0.83 (0.75–0.86)	0.81 (0.72–0.84)
**TP Inter-operator**	0.85 (0.78–0.87)	0.83 (0.75–0.84)	0.82 (0.7–0.82)	0.80 (0.71–0.80)
**SP Intra-rater**	0.91 (0.82–0.94)	0.89 (0.79–0.92)	0.86 (0.76–0.89)	0.84 (0.73–0.86)
**SP Inter-rater**	0.88 (0.79–0.91)	0.85 (0.75–0.89)	0.83 (0.72–0.87)	0.81 (0.69–0.85)
**SP Intra-operator**	0.85 (0.76–0.88)	0.82 (0.72–0.85)	0.82 (0.72–0.85)	0.76 (0.66–0.80)
**SP Inter-operator**	0.82 (0.73–0.85)	0.79 (0.69–0.82)	0.76 (0.66–0.80)	0.74 (0.64–0.78)

### 3.2 Assessing validity

**Fig. 2:**
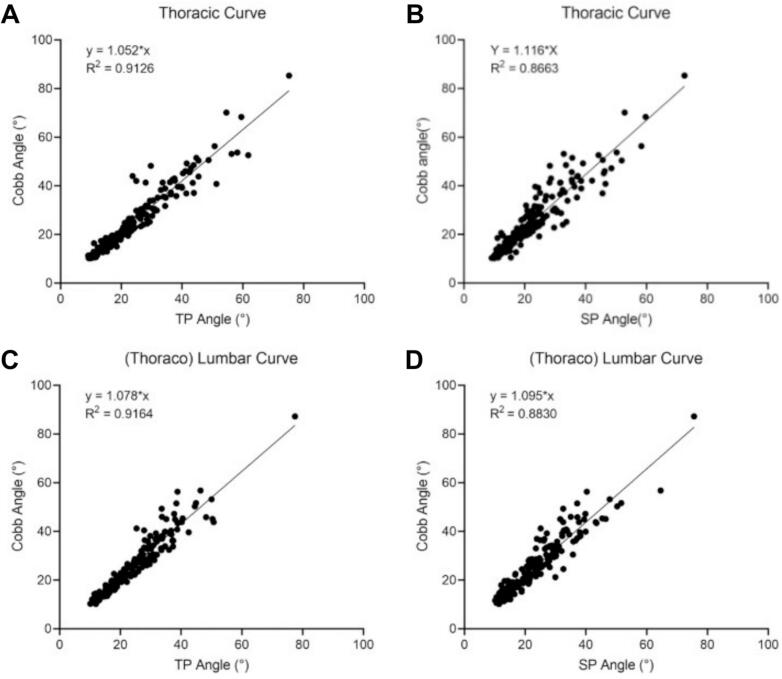
Correlation and regression analysis between TP/SP angle and cobb angle in thoracic and (thoraco) lumbar Curve. (A) TP angle vs. thoracic cobb Angle. (B) TP angle vs. (thoraco) lumbar cobb Angle. (C) SP angle vs. thoracic cobb angle. (D) SP angle vs. (thoraco) lumbar cobb angle.

Pearson linear regression analyses revealed robust correlations between standard Cobb angle measurements and alternative angular quantification methods across spinal curvature types. The consistency analysis of the UCA measurements based on the TP method showed superior concordance compared to the SP method, both for the main thoracic and (thoraco) lumbar curves (Fig. 2).

**Fig. 3:**
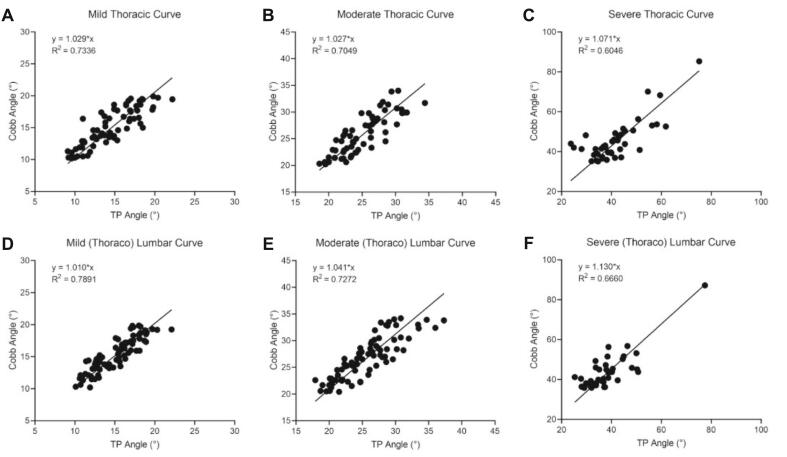
Correlation and regression analysis between TP angle and cobb angle stratified by curve severity in thoracic and (thoraco) lumbar curve. (A) Mild thoracic curve. (B) Moderate thoracic curve. (C) Severe thoracic curve. (D) Mild (thoraco) lumbar curve. (E) Moderate (thoraco) lumbar Curve. (F) Severe (thoraco) lumbar Curve.

**Fig. 4:**
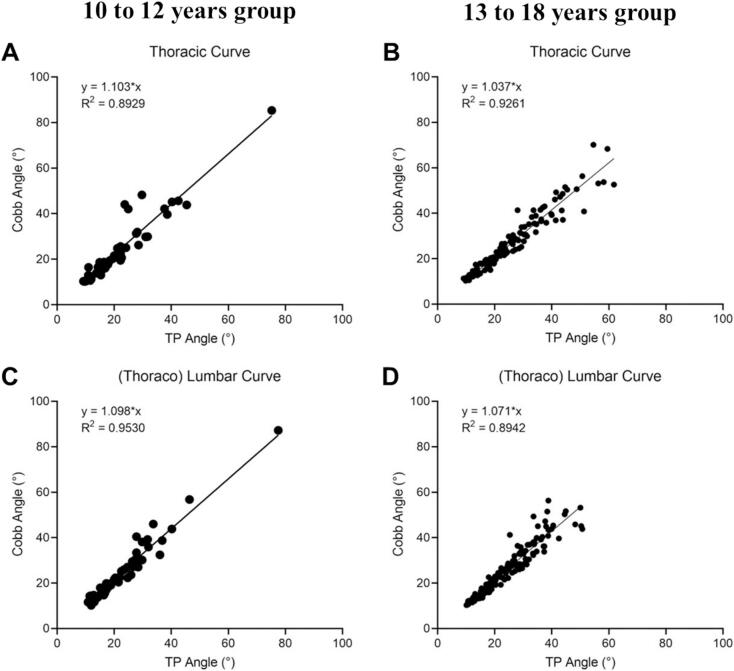
Correlation and regression analysis between TP angle and cobb angle stratified by age in thoracic and (thoraco) lumbar Curve. (A) Thoracic curve 10–12 years. (B) Thoracic curve 13–18 years. (C) (Thoraco) lumbar curve 10–12 years. (D) (Thoraco) lumbar curve 13–18 years.

**Fig. 5:**
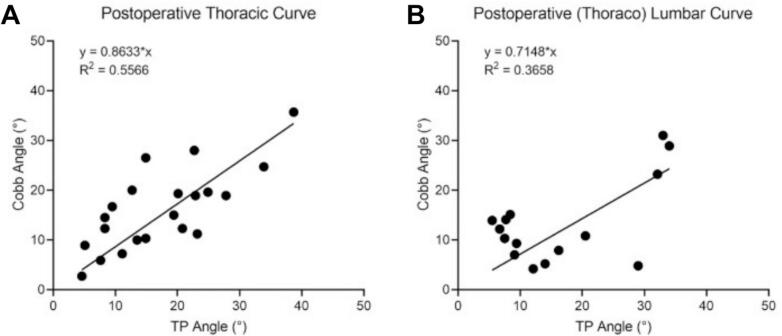
Correlation and regression analysis between TP angle and cobb angle with postoperative evaluation in thoracic and (thoraco) lumbar curve. (A) Postoperative thoracic curve. (B) Postoperative (thoraco) lumbar curve.

**Fig. 6:**
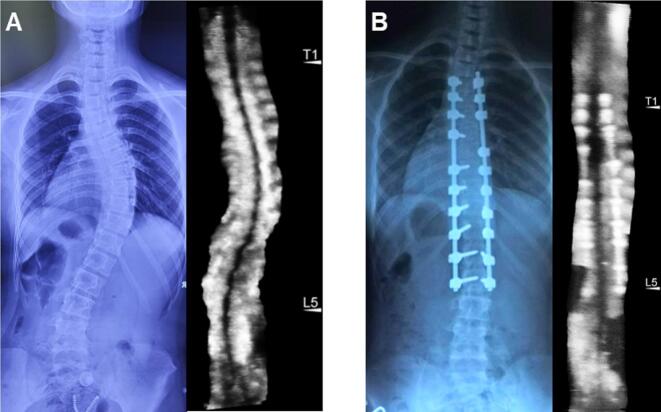
Comparative pre-operative and post-operative spinal ultrasound imaging in the same patient. (A) Pre-operative ultrasound scan. (B) Post-operative ultrasound scan.

The TP method was prioritized for subsequent validation analyses based on its superior test–retest reliability. Significant agreement between UCA and X-ray Cobb angles for the main thoracic curve and (thoraco) lumbar curve at different degrees of severity (R^2^ > 0.6), though comparative analysis revealed progressive concordance deterioration in severe deformities (Fig. 3). Age-stratified analysis confirmed maintained UCA-radiographic consistency for both thoracic and (thoraco) lumbar curves using TP protocols (R^2^ > 0.85) (Fig. 4). Postoperatively, ultrasound-radiographic agreement declined substantially (thoracic: R^2^ = 0.56; (thoraco) lumbar: R^2^ = 0.37), representing statistically significant reductions versus preoperative baselines (Fig. 5, Fig. 6).

### 3.3 Clinical application

**Fig. 7:**
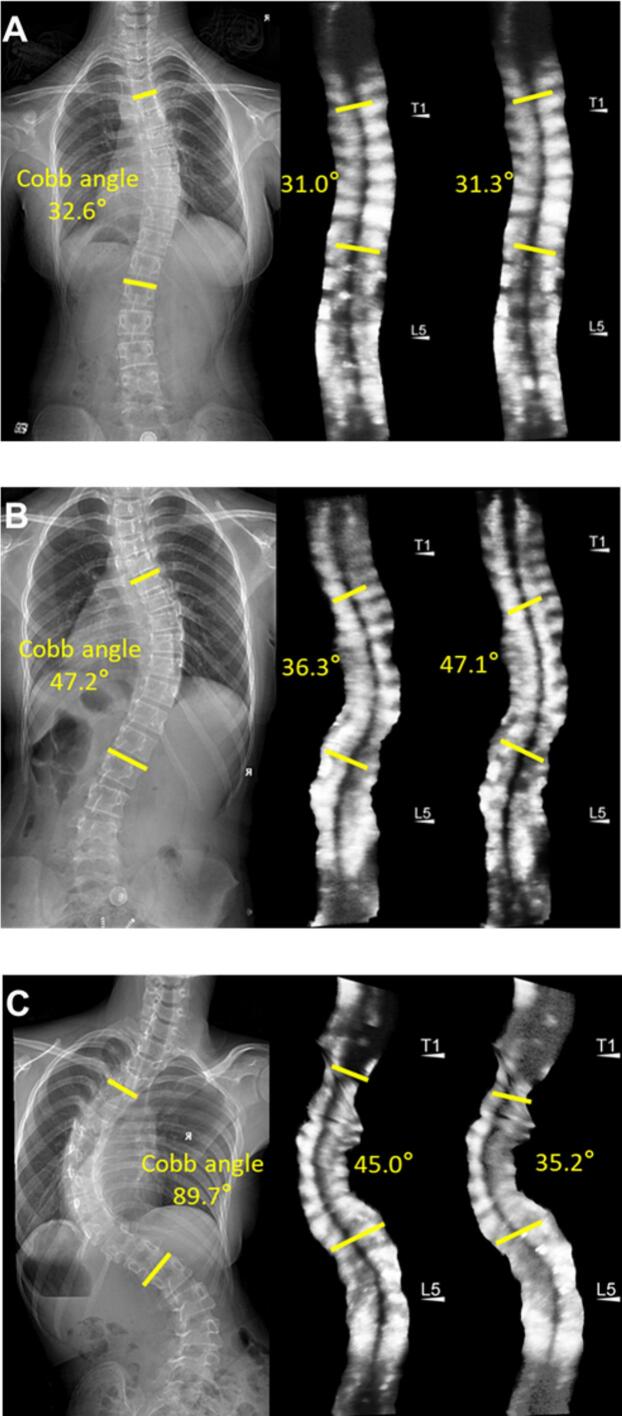
Summary of ultrasound imaging characteristics in AIS. (A) Successful initial and secondary scans. (B) Initial scan suboptimal, secondary scan successful. (C) Persistent imaging challenges despite multiple scanning attempts.

The mean ultrasound acquisition time per examination was 37.3 ± 6.8 s. High-quality images were successfully acquired in 81 % of patients (n = 186) during the initial scan. Suboptimal imaging outcomes occurred in 15 % of initial scans (n = 35), primarily due to anatomical variations and acoustic shadowing from razor back. Image quality optimization was accomplished through operator-guided protocol optimization tailored to anatomical variations during secondary scanning. Technical limitations persisted in 4 % of patients (n = 9) despite implementation of optimized scanning protocols (Fig. 7).

### 3.4 Factors influencing ultrasound measurements

Multivariate regression analysis revealed distinct biomechanical factors affecting ultrasound measurement accuracy across spinal regions. For thoracic curve quantification, thoracic kyphosis (B = 3.334, 95 % CI [0.736, 5.932], p = 0.012) and vertebral rotation (B = 1.862, 95 % CI [0.292, 3.431], p = 0.02) demonstrated significant predictive value. In (thoraco) lumbar assessments, vertebral rotation emerged as the dominant confounding factor (B = 2.209, 95 % CI [0.979, 3.440], p < 0.01). These results establish spinal morphological features necessitating protocol refinement for subsequent methodological development (Table 4, Table 5).

**Table 4:** Multivariate linear regression of determinants influencing ultrasound accuracy in thoracic curvature measurements.

**Variables**	**B**	**95 % CI (B)**		**S. E**	**β**	**t**	**P values**
		**lower**	**upper**				
**Thoracic kyphosis**	3.334	0.736	5.932	1.313	0.225	2.539	0.012*
**Vertebral rotation**	1.862	0.292	3.431	0.793	0.205	2.347	0.020*
**Obesity**	−1.016	−3.844	1.813	1.429	−0.060	−0.711	0.479
**Age**	−0.132	−0.368	0.103	0.119	−0.0097	−1.112	0.268
**Gender**	0.469	−1.170	2.109	0.828	0.051	0.567	0.572

Note: R^2^ = 0.100, adjusted R = 0.064.

**Table 5:** Multivariate linear regression of determinants influencing ultrasound accuracy in (thoraco) lumbar curvature measurements.

**Variables**	**B**	**95 % CI (B)**		**S. E**	**β**	**t**	**P Values**
		**lower**	**upper**				
**Thoracic kyphosis**	0.378	−1.544	2.301	0.973	0.031	0.389	0.698
**Vertebral rotation**	2.209	0.979	3.440	0.623	0.282	3.546	0.001*
**Obesity**	−0.0349	−2.617	1.920	1.148	−0.024	−0.304	0.762
**Age**	−0.022	−0.195	0.151	0.088	−0.020	−0.251	0.802
**Gender**	−0.372	−1.580	0.835	0.611	−0.049	−0.609	0.544

Note: R^2^ = 0.088, adjusted R = 0.058.

## 4 Discussion

As a progressive three-dimensional spinal deformity with established correlations to cardiopulmonary compromise and reduced quality of life [[1], [2], [3], [4], [5]], AIS remains dependent on radiographic Cobb angle measurements for diagnostic classification and surveillance [9,10]. This clinical concern regarding cumulative radiation exposure necessitates exploration of alternative modalities. 3-D ultrasound has emerged as a candidate technology, offering non-invasive, radiation-free, real-time visualization while providing accurate assessments. Nevertheless, the diagnostic reliability and measurement validity of this emergent modality across a heterogeneous AIS cohort. This investigation pursues dual objectives: prospectively evaluating the diagnostic efficacy and operational effectiveness of this modality through a standardized validation framework in heterogeneous AIS cohorts, while concurrently identifying confounding technical factors affecting measurement precision under clinical operating conditions.

Previous studies on the repeatability of both radiographic Cobb and ultrasound measurements in AIS patients have demonstrated high intra- and inter-rater/operator reliability [17,22,24,25]. Quantitative analysis established the clinometric adequacy of both TP and SP anchored ultrasound curvature assessments, with TP-based measurements demonstrating significantly enhanced intra- and inter-rater/operator consistency (ICC > 0.80) compared to SP-derived values (ICC > 0.70). The magnitude-dependent decline in ICC with increasing curvature severity may reflect anatomical complexities in severe scoliosis, such as pronounced vertebral rotation or rib humps, which obscure transverse process visualization [26,27]. Notably, the suboptimal reliability of SP may be attributable to inherent anatomical constraints and anisotropic echo patterns in ultrasound imaging, thereby amplifying operator dependence during probe repositioning maneuvers.

Systematic validation studies have established the UCA as a viable alternative for coronal plane deformity characterization, demonstrating excellent diagnostic concordance with radiographic Cobb measurements (R^2^ values from 0.76 to 0.99) [[17], [18], [19],^22^,^28^,^29^]. This investigation demonstrates UCA based on both TP and SP methods demonstrated strong consistency with radiographic Cobb angles. Furthermore, SP-derived measurements exhibited comparatively lower consistency than TP-based evaluations. This observation aligns with the methodological findings of Brink et al. [18], who similarly documented a greater magnitude of angular discrepancy in SP relative to the TP when compared against radiographic Cobb criteria. This potentially attributable to SP heightened sensitivity to paravertebral soft tissue artifacts during landmark identification. In contrast, transverse processes offer morphological advantages through their broader acoustic footprint and predictable spatial configuration, facilitating more reliable anatomic landmark acquisition in multiplanar scanning protocols.

Importantly, the UCA-Cobb angle correlation demonstrated significant heterogeneity across scoliosis severity subgroups (mild/moderate/severe), reflecting fundamental shifts in biomechanical coupling dynamics between osseous landmarks and spinal deformity patterns at different curvature magnitudes. In mild-to-moderate curves (Cobb ≤ 35°), robust correlations were observed, with R^2^values ranging from 0.70 to 0.80 across both thoracic and (thoraco) lumbar regions. A notable correlation attenuation occurred in severe deformities (Cobb > 35°), most pronounced in (thoraco) lumbar segments (R^2^ = 0.66). This severity-dependent attenuation originates from the pathophysiological evolution of spinal deformity, primarily mediated through progressive vertebral rotation mechanics and multiaxial load redistribution in advanced stages. Thoracic cage deformation and nonlinear coupling between sagittal-coronal plane deformities further compromise surface-based measurement fidelity in severe torsion states. Paradoxically, while severe deformities characterized by rib hump prominence and coronal plane shoulder asymmetry render scoliosis readily identifiable through standard clinical inspection, ultrasound-based screening maintains diagnostic efficacy in this population. Given this, spinal ultrasound proves to be highly effective as a screening tool, even in the presence of more severe deformities. Furthermore, the consistency across different age groups was generally good. Its ability to identify obvious clinical manifestations of scoliosis supports its valuable role in early detection and routine screening.

Postoperative analysis revealed marked attenuation in UCA-Cobb angle correlation compared to preoperative baseline measurements, with thoracic curve agreement declining from R^2^ = 0.91 to 0.56 and (thoraco) lumbar correlation decreasing from R2 = 0.92 to 0.37. The inherent limitations of ultrasonography hinder its ability to overcome postoperative paraspinal scar tissue formation and implant-induced acoustic shadowing, thereby significantly compromising the sonographic visualization of transverse process landmarks. [30]. This study reveals accuracy limitations in postoperative spinal ultrasonography, necessitating chemical and physical technological advancements to enhance ultrasound technology for improved clinical utility.

Spinal ultrasound demonstrates clinically viable scan efficiency (mean 37.3 ± 6.8 s) with first-attempt success rates exceeding 80 % for diagnostic-quality image acquisition. This efficiency gain likely reflects advancements in transducer ergonomics and standardized landmark identification workflows. While acoustic shadowing from razor back caused technical limitations in 15 % of cases, operator adaptation resolved most issues during secondary scans. The residual 4 % of challenging cases predominantly involved severe rotational deformities, corroborating the findings of Brink et al. [18] regarding ultrasonographic limitations in complex three-dimensional spinal deformities.

Biomechanical analyses suggest that significant axial vertebral rotation (AVR) combined with a Cobb angle ≥ 25° significantly compromises sonographic reliability through vertebral shadowing effects, resulting in clinically significant divergence between radiographic and ultrasonographic quantification [30]. Multivariate regression modeling identified vertebral rotation as a key confounding factor influencing the accuracy of thoracic and (thoraco) lumbar angle measurements (B = 1.862–2.209, p < 0.05), mediated through rotational artifact generation that disrupts transverse process sonographic registration. Additionally, thoracic kyphosis (B = 3.334, p < 0.05) emerged as a critical modulator of thoracic region measurement variability due to ultrasound beam angulation constraints in altered sagittal alignments. These biomechanical interactions necessitate rigorous preoperative evaluation of rotational and sagittal plane deformity when interpreting ultrasonographic data, particularly in multiaxial deformity configurations where compounded vertebral torsion and rib cage distortion exceed conventional sonographic resolution thresholds.

The limitations of study methodological primarily derive from its single-center cohort design, which may introduce selection. Furthermore, the absence of longitudinal surveillance data precludes definitive assessment of ultrasonographic sensitivity to deformity progression dynamics. The constrained statistical power of single-center postoperative cohort analyses necessitates both multicenter collaboration and the establishment of extended follow-up cohorts to achieve reliable detection of subtle biomechanical parameters in spinal implant evaluations.

This validation study confirms TP-anchored 3-D ultrasound as clinically viable non-ionizing modality for assessing mild-to-moderate AIS curves, demonstrating diagnostic concordance with X-ray. While curvature-dependent diagnostic accuracy decay is observed due to ultrasound beam penetration constraints in complex spinal topography, operational efficiency persists through standardized sub-50-second acquisition protocols and 85 % first-attempt success rates. Multivariate regression analysis confirmed vertebral rotation and thoracic kyphosis as primary determinants of ultrasonographic measurement variance. These findings validate its utility for routine screening, while targeted technical refinements may expand applicability to severe/postoperative cases.

## 5 Conclusion

TP-based 3D ultrasound provides accurate, radiation-free spinal assessments in mild-to-moderate AIS, showing strong agreement with X-ray. Vertebral rotation and thoracic kyphosis were identified as critical factors limiting measurement accuracy. Despite reduced efficacy in complex deformities, the operational efficiency of the method, diagnostic precision, and radiation-free safety profile support its broad applicability for adolescent scoliosis screening.

## CRediT authorship contribution statement

**Xinyu Yang:** Formal analysis, Data curation, Conceptualization. **Derun Di:** Formal analysis, Data curation, Conceptualization. **Yanhong Lv:** Resources, Formal analysis, Data curation. **Zhe Liu:** Formal analysis, Data curation, Conceptualization. **Xiaoxiong Wang:** Formal analysis, Data curation, Conceptualization. **Xinzhi Zhang:** Formal analysis, Data curation, Conceptualization. **Rongkun Xu:** Formal analysis, Data curation, Conceptualization. **Luoran Wang:** Formal analysis, Data curation, Conceptualization. **Yuze Wang:** Writing – review & editing, Data curation, Conceptualization. **Suomao Yuan:** Project administration, Investigation, Conceptualization. **Yonghao Tian:** Supervision, Software, Resources. **Qiang Yang:** Writing – review & editing, Writing – original draft, Project administration. **Xinyu Liu:** Writing – review & editing, Project administration, Funding acquisition. **Lianlei Wang:** Writing – review & editing, Investigation, Funding acquisition.

## Ethics approval

This prospective study was approved by the research Ethics Committee at Qilu Hospital of Shandong University (KYLL-2021(KS)-055)

## Funding

This research was supported in part by the National Nature Science Foundation (81874022, 82,172,483 and 82102522), National Key R&D Program of China (2023YFC2509700), Shandong Province Taishan Scholar Project (tsqn202211317 and tstp20231247), Key R&D Project of Shandong Province (2022CXGC010503 and 2022ZLGX03), Shandong Natural Science Foundation (ZR202102210113) and National High Level Hospital Clinical Research Funding (2022-PUMCH-D-004).

## Declaration of competing interest

The authors declare that they have no known competing financial interests or personal relationships that could have appeared to influence the work reported in this paper.
